# IP-10 response to RD1 antigens might be a useful biomarker for monitoring tuberculosis therapy

**DOI:** 10.1186/1471-2334-11-135

**Published:** 2011-05-19

**Authors:** Basirudeen Syed Ahamed Kabeer, Alamelu Raja, Balambal Raman, Satheesh Thangaraj, Marc Leportier, Giuseppe Ippolito, Enrico Girardi, Philippe Henri Lagrange, Delia Goletti

**Affiliations:** 1 Department of Immunology, Tuberculosis Research Centre (ICMR), Tamil Nadu, Chennai, India; 2 Department of Clinical Research, Tuberculosis Research Centre (ICMR), Mayor V.R. Ramanathan Road, Chetpet, Chennai -- 600 031, Tamil Nadu, India; 3 Biomérieux, Research & Development Immunoassays, Chemin de l'Orme, Marcy L'Etoile, France; 4 Scientific Direction, Lazzaro Spallanzani National Institute for Infectious Diseases (INMI), Rome, Italy; 5 Department of Epidemiology and Preclinical Research, INMI, Rome, Italy; 6 Microbiology Service, Saint Louis Hospital, Paris, France; 7 Translational Research Unit, Department of Epidemiology and Preclinical Research, (INMI), Rome, Italy

## Abstract

**Background:**

There is an urgent need of prognosis markers for tuberculosis (TB) to improve treatment strategies. The results of several studies show that the Interferon (IFN)-γ-specific response to the TB antigens of the QuantiFERON TB Gold (QFT-IT antigens) decreases after successful TB therapy. The objective of this study was to evaluate whether there are factors other than IFN-γ [such as IFN-γ inducible protein (IP)-10 which has also been associated with TB] in response to QFT-IT antigens that can be used as biomarkers for monitoring TB treatment.

**Methods:**

In this exploratory study we assessed the changes in IP-10 secretion in response to QFT-IT antigens and RD1 peptides selected by computational analysis in 17 patients with active TB at the time of diagnosis and after 6 months of treatment. The IFN-γ response to QFT-IT antigens and RD1 selected peptides was evaluated as a control. A non-parametric Wilcoxon signed-rank test for paired comparisons was used to compare the continuous variables at the time of diagnosis and at therapy completion. A Chi-square test was used to compare proportions.

**Results:**

We did not observe significant IP-10 changes in whole blood from either NIL or QFT-IT antigen tubes, after 1-day stimulation, between baseline and therapy completion (p = 0.08 and p = 0.7 respectively). Conversely, the level of IP-10 release to RD1 selected peptides was significantly different (p = 0.006). Similar results were obtained when we detected the IFN-γ in response to the QFT-IT antigens (p = 0.06) and RD1 selected peptides (p = 0.0003). The proportion of the IP-10 responders to the QFT-IT antigens did not significantly change between baseline and therapy completion (p = 0.6), whereas it significantly changed in response to RD1 selected peptides (p = 0.002). The proportion of IFN-γ responders between baseline and therapy completion was not significant for QFT-IT antigens (p = 0.2), whereas it was significant for the RD1 selected peptides (p = 0.002), confirming previous observations.

**Conclusions:**

Our preliminary study provides an interesting hypothesis: IP-10 response to RD1 selected peptides (similar to IFN-γ) might be a useful biomarker for monitoring therapy efficacy in patients with active TB. However, further studies in larger cohorts are needed to confirm the consistency of these study results.

## Background

The T cell-based assays using region of difference (RD)1 antigens, such as early secreted antigenic target, 6 kDa (ESAT-6), and culture filtrate protein, and 10 kDa (CFP-10), have an evolving niche in detecting *Mycobacterium tuberculosis *infection. The RD1 sequence is missing from *M. bovis *Bacille Calmette-Guerin (BCG), and this omission makes these antigens more specific for *M. tuberculosis *infection diagnosis than the purified protein derivative (PPD) [1-3]. The two RD1 antigens, ESAT-6 and CFP-10, have been shown to induce strong Interferon (IFN)-γ response during short term incubation *in vitro *[3,4]. Based on this principle, there are two commercial kits available for diagnosing tuberculosis (TB) infection, the QuantiFERON-TB GOLD In-Tube^® ^(QFT-IT) (Cellestis Ltd., Carnegie, Australia) and T-SPOT*.TB*^® ^(Oxford Immunotec, Abingdon, UK). The performance of these assays is extensively reviewed [5-8] indicating that they are at least as sensitive as the tuberculin skin test (TST) in detecting latent TB infection (LTBI) and active TB cases.

Animal and human studies have shown a relationship between the magnitude of IFN-γ responses and mycobacterial bacillary load [9,10]. It has, therefore, been postulated that a decrease in the magnitude of IFN-γ responses to *M. tuberculosis *specific antigens might be used as a biomarker of treatment response [11]. However, studies using serial QuantiFERON-TB Gold tests or IFN-γ ELISPOT assay in adults (performed during treatment of either LTBI [12-17] or active TB disease [9,18-26]) in various settings have shown conflicting results, with IFN-γ responses decreasing [14,15,17-20,22,26], increasing [12,16] or remaining almost unchanged [13,21,23-25] in response to treatment.

We developed an *in vitro *IFN-γ immune diagnostic assay for active TB disease, the novelty of which consists of the use of multiepitopic RD1 peptides selected by computational analysis [27-29]. IFN-γ response to these RD1 selected peptides can be detected in individuals with ongoing *M. tuberculosis *replication (such as during active TB disease and/or recent TB infection) and has been shown to significantly decrease in Human Immunodeficiency Virus (HIV) uninfected [17,18] or infected individuals [30] during TB prophylaxis and therapy.

It has recently been shown (by others and us) that IFN-γ-inducible protein (IP)-10 is a potential diagnostic marker [31-37]. An enzyme linked immunosorbant assay (ELISA), which measures levels of IP-10 in whole blood after overnight stimulation with TB Antigens of the QFT-IT format (hereinafter referred to as "QFT-IT antigens"), has demonstrated to have similar sensitivity for detecting active TB compared to QFT-IT and to the IFN-γ assay based on RD1 selected peptides in HIV-uninfected subjects [32]. Higher sensitivity in diagnosing TB cases for both IP-10-based assays has been described in HIV-infected subjects compared to the corresponding IFN-γ-based tests [34,36,38]. However, to our knowledge, the kinetics of IP-10 secretion in response to QFT-IT antigens before and after treatment has never been investigated.

Azzurri et al [39] have described a decline in the levels of IP-10 in plasma after successful anti-TB treatment. Furthermore, the previous observations have demonstrated that patients with active TB had higher IP-10 levels in the NIL tube (unstimulated whole blood culture tube used in the QFT-IT assay) compared to healthy controls [40].

Thus, in this exploratory study involving 17 enrolled subjects (a subgroup of the 41 HIV-uninfected individuals previously described [33]), we evaluated whether or not IP-10 can be a good biomarker for monitoring TB therapy. Therefore, we assessed the changes of IP-10 levels from the NIL and QFT-IT antigen tubes and RD1 selected peptides stimulated whole blood in patients with active TB disease at the time of TB diagnosis and after successful specific treatment. IFN-γ response to QFT-IT antigens and RD1 selected peptides were evaluated as controls.

## Methods

### Study subjects

This study has been approved by the Institutional Ethical Committee of the Tuberculosis Research Centre, Chetput, Chennai TRC-IEC (No: 2006005) and written consent was obtained from each study subject. Study subjects were prospectively recruited from the Revised National Tuberculosis Control Program (RNTCP) centers from April, 2007 to March, 2008. Subjects who were diagnosed as pulmonary TB patients at the RNTCP center were assessed for the study. Individuals with a previous history of TB, who had undergone TST in the past 16 months, who had HIV infection, silicosis, end stage renal disease, leukemia/lymphoma or who were undergoing immunosuppressive therapy were excluded from the study.

After registering, the eligible subjects underwent radiological examinations, and three sputum samples were collected from each. The collected sputum samples were processed [41], stained for acid fast bacilli (AFB) microscopy by Ziehl-Neelsen method and cultured in Lowenstein Jensen (BioMérieux Inc., Marcy I'Etoile, France) and in liquid MP BacT medium (BioMérieux Inc). The presence of *M. tuberculosis *in the positive culture samples was further confirmed by Gen-probe based PCR (BioMérieux Inc., Marcy I'Etoile, France) method. Therefore, active TB was defined as microbiologically confirmed if the criteria stated above was fulfilled. Conversely, patients were classified as having "clinical TB" if the diagnosis was based on clinical and radiologic criteria (after excluding other diseases) including appropriate response to anti-TB therapy.

Blood was drawn from all the recruited study subjects for a total blood count, HIV testing and IFN-γ- and IP-10-based assays. All subjects were treated with a standard regimen of rifampicin, isoniazid, ethambutol and pyrazinamide for 2 months and then, if the clinical conditions and chest X-rays improved and AFB sputum conversion occurred, rifampicin and isoniazid were continued for an additional 4 months [42]. At the end of six months, sputum samples and blood samples were collected once again and assessed for their response to treatment and to the in vitro test, respectively.

### Stimulation of whole blood with QFT-IT antigens

A commercial QFT-IT assay (Cellestis) was used to evaluate the QFT-IT antigen-specific IFN-γ and IP-10 secretion. Briefly, one ml of blood was taken into each of the three tubes: pre-coated either with QFT-IT antigens, phytohemaglutinin for the positive control or no antigen for the negative control (NIL). The blood samples were drawn between 10 and 11 am and taken to the laboratory within 2 hours of phlebotomy. The tubes were incubated for 16-24 hours at 37°C and plasma were collected after centrifugation and stored at 4°C until tested.

### RD1 selected peptides and whole blood cultures

The selection of Human Leukocyte Antigens (HLA)-class II restricted epitopes of ESAT-6 and CFP-10 *M. tuberculosis *proteins was performed by a quantitative, implemented, HLA peptide-binding motif analysis as previously described for ESAT-6 [27-29]. Peptides were synthesized as free amino acid termini using Fmoc chemistry (ABI, Bergamo, Italy). All synthetic peptides were purified by reverse-phase chromatography to have at least 90% purity. Sequence and purity were confirmed by mass spectrometry and analytical reverse-phase chromatography [27]. Lyophilized peptides were diluted in Dimethyl Sulfoxide (DMSO) at stock concentrations of 10 mg/mL for each peptide and stored at -80°C. RD1 selected peptides were used as follows: a pool of the two ESAT-6 peptides (at 10 μg/mL each) and a pool of the three CFP-10 peptides (at 2 μg/mL each). DMSO was used as a negative control at 10 μg/mL. The whole blood test was carried out as described [33,36]. Briefly, aliquots of 0.5 ml per well of heparinised blood in monoplicate were seeded in a 48-well plate and stimulated with or without RD1 selected peptides, as described above. Samples were then incubated for 16-24 hours at 37°C in the presence of 5% CO_2 _when 100 μl of plasma was harvested.

Indian collaborators were provided with RD1 selected peptides from the same batch, detailed protocol and personal training by INMI's laboratory staff. Inter-site communication was present throughout the study to solve any potential problems. Clinicians were blinded to the laboratory test results and laboratory staff was blinded to the status of the patients.

### IP-10 assay

The IP-10 levels were measured in the plasma samples using human IP-10 ELISA Set (R&D Sysytems, USA) as per the manufacturer's instructions [33,36]. To detect the chemokines, plasma was diluted 1:10 as a starting dilution. Further dilutions were performed when necessary. The IP-10 data from QFT-IT antigens or RD1 peptides stimulated culture provided in the text and figures are reported after subtracting the respective unstimulated controls, which is either the whole blood culture incubated with the same concentration of DMSO used to dissolve the peptides for the RD1 selected peptides stimulated conditions [33,36] or the NIL tube for the QFT-IT antigens.

### Measurement of IFN-γ

The QFT-IT ELISA (Cellestis) was performed to measure the IFN-γ levels in the plasma samples following the manufactures instructions (Cellestis Ltd., Victoria, Australia). The test results were interpreted using software supplied by the manufacturer (Cellestis Ltd., Victoria, Australia). Values above 10 IU/ml were considered as equal to 10 IU/ml, as indicated by the manufacturers. The IFN-γ data from QFT-IT antigens or RD1 peptides stimulated culture provided in the text and figures are reported after subtracting the respective unstimulated controls, which is either the whole blood culture incubated with the same concentration of DMSO used to dissolve the peptides for the RD1 peptides stimulated conditions [33,36] or the NIL tube for the QFT-IT antigens.

### Longitudinal analysis of the IP-10 and IFN-γ data

A longitudinal analysis of the IP-10 and IFN-γ data was made considering the highest IP-10 response to either ESAT-6 or CFP-10 selected peptides per single patient at both baseline and end of treatment.

### Eligibility criteria for the study

Enrolled patients were defined as "eligible" if the experimental data at both, baseline and after 6 months treatment were available. Data analysis was performed only on the subjects that met the eligibility criteria.

### Statistical analysis

The main outcome of the study on IP-10 and IFN-γ production in response to QFT-IT antigens and RD1 selected peptides was expressed as continuous (IU/ml) or dichotomous (positive/negative) measures. For continuous measures, the median and interquartile range (IQR) was calculated. A non-parametric Wilcoxon signed-rank test was used for paired comparisons. Differences were considered significant at p values ≤ 0.05.

For dichotomous measures, chi square was used. For pair-wise comparisons, differences were considered significant at p values ≤ 0.05. SPSS v 14 for Windows (SPSS Italia Srl, Bologna, Italy) and Prism 4 software (GraphPad Software 4.0, San Diego, CA, USA) were used in the analysis.

## Results

### Characteristics of the subjects included in the study

As previously described [33], a total of 41 HIV-uninfected individuals were assessed for this report. However, only 17 subjects met the eligibility criteria for the study. The median duration of TB treatment was 6 months (IQR: 6.0-6.1).

The median age of the eligible 17 subjects was 32 years and 9 of them were males (Table 1). Regarding the microbiological data, 12 (71%) were positive for AFB sputum microscopy and 16 (94%) were positive to sputum culture. In the remaining 1 subject, diagnosis was made based on clinical criteria.

**Table 1:** Demographic and clinical characteristics of the subjects enrolled in the study

Parameter	Total N. 17
**Age, mean in years (IQR)**	32 (25-52)
**Sex**	
Male, Number (%)	9 (52.9)
**Smear Positivity, Grade Number (%)**	
1+	6 (35.3)
2+	4 (23.5)
3+	2 (11.8)
0	5 (29.4)
**Culture Results, Number (%)**	
Positive	16 (94.1)
Negative	1 (5.9)
**TB Severity, Number (%)**	
Mild/Moderate	12 (70.6)
Severe	5 (29.4)

**Abbreviations**: IQR: interquartile range; TB: tuberculosis; IP: inducible protein; IFN: interferon; RD: region of difference.

All 17 subjects completed the anti-tuberculosis therapy by end of six months. They were negative for AFB sputum microscopy after 2 months of treatment and at therapy completion. Radiological examination also confirmed their healthy status.

### Longitudinal analysis of IP-10 secretion to NIL and DMSO samples in patients with active TB who were followed until therapy completion

**Figure 1:**
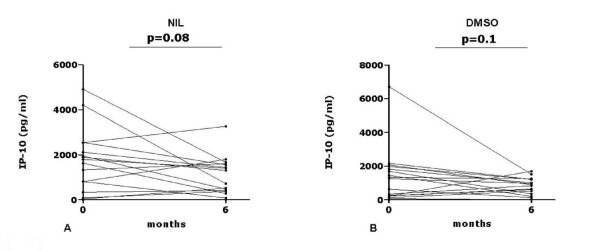
**Longitudinal analysis of IP-10 levels in NIL and DMSO conditions after 1-day culture in samples from patients with active TB who were followed until specific therapy completion**. IP-10 levels in the NIL (**A**) and DMSO (**B**) conditions after 1-day culture in samples from patients with active TB evaluated before therapy (T0) and at therapy completion (T6). No significant changes were recorded, as indicated by the p values. **Abbreviations**: IP: interferon inducible protein. DMSO: Dimethyl Sulfoxide.

Azzurri et al [39] have described a decline in the IP-10 levels in plasma after successful anti-TB treatment. Furthermore, the previous observations have demonstrated that patients with active TB had higher IP-10 levels in NIL tubes when compared to healthy controls [40]. Assuming that successful treatment reverts the IP-10 levels in the unstimulated cultures, we evaluated the IP-10 level in the plasma from NIL tubes and DMSO whole blood cultures, after 1-day stimulation, in the patients at the time of TB diagnosis (T0) and at therapy completion (T6). As shown in Figure 1, no significant changes were observed when comparing the IP-10 levels in the NIL tubes at T0 (median: 1630; IQR: 212-2330) to T6 (median: 722; IQR: 326-1581) (p = 0. 0833) (Figure 1A) or comparing the IP-10 levels in the DMSO cultures at T0 (median: 1250; IQR: 221-1898) to T6 (median: 830; IQR: 269-1206) (p = 0.1148) (Figure 1B). These data indicate the absence of significant IP-10 changes in the unstimulated culture in this longitudinal analysis. Therefore, we evaluated the changes in QFT-IT and RD1 selected peptides stimulated whole blood.

### Longitudinal analysis of IP-10 secretion in response to the QFT-IT antigens in patients with active TB who were followed until therapy completion: comparison with IFN-γ results

**Figure 2:**
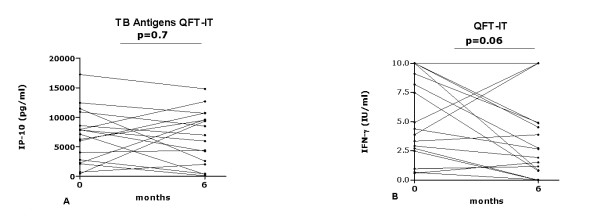
**Longitudinal analysis of IP-10 secretion in response to the QFT-IT antigens in patients with active TB who were followed until specific therapy completion: comparison with IFN-γ results**. IP-10 response to the QFT-IT antigens (**A**) and IFN-γ response (QFT-IT) (**B**) in patients with active TB evaluated before therapy (T0) and at therapy completion (T6). No significant changes were recorded, as indicated by the p values. **Abbreviations**: IP: interferon inducible protein; IFN: interferon; IU: international units.

The level of IP-10 secretion in response to the QFT-IT antigens did not significantly change from the time of TB diagnosis (T0) (median: 7137 pg/ml; IQR: 2527-9756) to the end of treatment (T6) (median: 6969 pg/ml; IQR: 2299-10148) (p = 0.7) (Figure 2A) Using the cut-off point of 698 pg/ml, previously found by ROC analysis in the same Indian setting [35] (Table 2), 16 out of 17 subjects (94.1%) scored positive for IP-10 at the time of TB diagnosis (Table 3, Figure 2A). At therapy completion, the one subject who scored negative to IP-10 turned positive and three subjects who scored positive at enrolment became negative (Table 3). Hence, the number of IP-10 positive subjects at the end of treatment was (14/17, 82.3%) (Table 3).

**Table 2:** IP-10 and IFN-γ cut-off points employed for the assays

	**IP-10**		**IFN-γ**	
	
**Antigen**	**IP-10 (pg/ml)**	**Cut-off point provided by:**	**IFN-γ (IU/ml)**	**Cut-off point provided by:**
QFT-IT antigens*	698	ROC analysis [36]	0.35	[company, 44]
RD1 selected peptides	350	ROC analysis [36]	0.57	ROC analysis [33]

**Abbreviations**: *antigens used in QuantiFERON TB Gold In tube test format; TB: tuberculosis; IP: inducible protein; IFN: interferon; RD: region of difference; ROC: Receiver Operator Characteristics.

**Table 3:** Serial response to the IP-10-based and IFN-γ-based assays in patients with active TB

	QFT-IT antigens			RD1 selected peptides IFN-γ cut-off 0.57 IU/ml		
	Time points			Time points		
	**T0**	**T6**	***p *value**	**T0**	**T6**	***p *value**
** IP-10** **Positive over total (%)**	16/17 (94.1)	14/17 (82.3)	0.6	16/17 (94.1)	7/17 (41.1)	0.002
** IFN-γ** **Positive over total (%)**	17/17 (100)	14/17 (82.3)	0.2	16/17 (94.1)	7/17 (41.1)	0.002
** IP-10** **N of reversion over total (%)**	-	3/17 (17.6)		-	10/17 (58.8)	
** IP-10 N of conversion over total (%)**	-	1/17 (5.8)		-	0/17 (0)	
** IFN-γ** **N of reversion over total (%)**	-	3/17 (17.6)		-	9/17 (52.9)	
** IFN- γ** **N of conversion over total (%)**	-	0/17 (0)		-	0/17 (0)	

**Footnotes**: TB: tuberculosis; QFT-IT: QuantiFERON TB Gold In tube; IP: inducible protein; IFN: interferon; RD: region of difference; N: number; T0: baseline; T6: end of treatment.

IFN-γ secretion was tested as a control. The level of IFN-γ secretion in response to QFT-IT did not significantly change between the baseline (median: 4.38 IU/ml; IQR: 2.58-10.35) and therapy completion (median: 2.66 IU/ml; IQR: 0.82-4.89) (p = 0.7) (Figure 2B). Using the commercial cut-off value of 0.35 IU/ml (Table 2), 17 out of 17 subjects (100%) were positive to QFT-IT. At therapy completion, 3 subjects turned negative. The proportion of positive responders between baseline and therapy completion (14/17, 82.3%) was not statistically significant (p = 0.2) (Table 3).

### Longitudinal analysis of IP-10 secretion in response to the RD1 selected peptides in patients with active TB who were followed until therapy completion: comparison with the IFN-γ results

**Figure 3:**
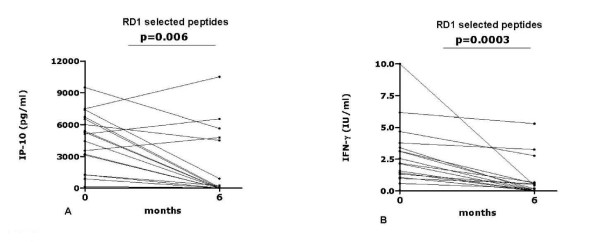
**Longitudinal analysis of IP-10 secretion in response to the RD1 selected peptides in patients with active TB who were followed until specific therapy completion: comparison with the IFN-γ results**. IP-10 (**A**) and IFN-γ response (**B**) to the RD1 selected peptides in patients with active TB evaluated before therapy (T0) and at therapy completion (T6). Significant changes were recorded, as indicated by the p values. **Abbreviations**: IP: interferon inducible protein; IFN: interferon; IU: international units.

When considering the highest IP-10 response to either ESAT-6 or CFP-10 selected peptides per single patient, the IP-10 secretion was significantly higher at the time of diagnosis (median: 5116 pg/ml; IQR: 2207-7063) than at therapy completion(T6) (median: 73 pg/ml; IQR: 0-5222) (p = 0.0060) (Figure 3A) Using the cut-off point of 350 pg/ml, previously found by ROC analysis [38] (Table 2), 16 out of 17 subjects (94.1%) scored positive for IP-10 at the time of TB diagnosis. At therapy completion, 9 subjects turned negative, whereas the individual who scored negative at enrolment was still negative after therapy completion. Therefore the proportion of positive responders significantly differed between baseline and therapy completion (7/17, 41.1%) (p = 0.002) (Table 3).

IFN-γ secretion was tested as a control. When considering the highest IFN-γ response to either ESAT-6 or CFP-10 selected peptides for single patient, the IFN-γ secretion was significantly higher at the time of diagnosis (median: 2.56 IU/ml; IQR: 1.20-4.18) than at therapy completion (median: 0.42 IU/ml; IQR: 0.02-1.72) (Figure 3B) (p = 0.0003). With the cut-off value of 0.57 IU/ml, previously found by ROC analysis [32] (Table 2), 16 out of 17 subjects (94.1%) scored positive in response to RD1 selected peptides at the time of TB diagnosis. At therapy completion, 9 subjects turned negative, whereas the individual who scored negative at enrolment was still negative after therapy completion. Therefore, the proportion of positive responders significantly differed between baseline and therapy completion (7/17, 41.1%) (p = 0.002) (Table 3).

When we used the same cut-off value used by the QFT-IT (0.35 IU/ml), no change was found in the score of RD1 selected peptides responders at the time of TB diagnosis. At the end of treatment, 7 subjects (instead of 9) turned negative, and the individual who scored negative at enrolment was still negative after therapy completion. The proportion of positive responders was still significantly different between baseline and therapy completion (9/17, 52.9%) (p = 0.02).

The changes in the secretion of IFN-γ or IP-10 in response to QFT-IT antigens or RD1 selected peptides did not depend on the grade of smear positivity, sputum culture positivity or severity of TB disease based on the chest X-ray results at the time of recruitment (data not shown).

## Discussion

In this exploratory study, we demonstrated (for the first time to our knowledge) that the IP-10 secreted response to selected RD1 peptides decreases during specific treatment in patients with active TB. A significant quantitative decrease in the level of IP-10 in response to the RD1 selected peptides was found between the baseline and end of TB treatment accompanied by a significant decrease in the positive rate of the test. Similar results were obtained by the detection of IFN-γ, confirming our previous reports [18,30]. Differently, the IP-10 response to the QFT-IT antigens did not significantly change by either quantitative or qualitative analysis. Interestingly, when considering the IFN-γ data, the quantitative responses to QFT-IT decreased, although not significantly, from baseline to the end of TB treatment whereas no change was found in the proportion of responders.

Previous studies have reported a decline in the levels of IP-10 in plasma after successful anti-TB treatment [39] and other earlier observations have demonstrated that patients with active TB have higher IP-10 levels in the plasma of unstimulated culture when compared to controls [40,43]. However in this study we were unable to confirm these data, probably due to the small number of patients analyzed.

The earlier studies conducted to assess the secretion of IFN-γ in response to QFT-IT ended up with conflicting results among those with active TB. While some of the studies reported significantly reduced IFN-γ secretion at the time of therapy completion compared to the baseline [9,18-20,22,26,30], other reports showed unchanged or minimal IFN-γ secretion upon effective therapy [21,23-25]. The probable reasons for this controversy might be due to several factors including re-infection, persistent infection, persistent exposure to mycobacteria, and possible maintenance of the circulating pool of effector memory T cells, rather than technical factors [7].

IP-10 secretion was elevated in active TB patients after stimulation with *M. tuberculosis *antigens [31-36,40,43,44]. However, similar to IFN-γ, the IP-10 secretion in response to the QFT-IT antigens did not change upon effective therapy. This is not unexpected, as IP-10 secretion is mainly induced by antigen-specific IFN-γ secreting T cells. Furthermore, previously [33,33], we found a good correlation between the level of IFN-γ and IP-10 in subjects with TB infection. Interestingly, in contrast to QFT-IT antigens, we found decreased IFN-γ and IP-10 levels after overnight stimulation with selected RD1 peptides after successful therapy.

The difference in the levels of IP-10 and IFN-γ secretion between QFT-IT antigens and selected RD1 peptides might be related to the amount and the composition of epitopes covered by the peptides used in the two different tests. For example, the peptides employed in the QTF-IT cover the whole CFP-10 and ESAT-6 intact proteins (in addition to having a peptide from TB7.7 from the RD11 region) [41] whereas the peptides used in our assay are few and selected in order to be highly immunogenic [27,28]. The response is mediated by the CD4+ T cells with an effector memory phenotype, as previously shown [45]. Based on our data, this oligoclonal response (more than polyclonal against all RD1 epitopes) appears to be a sensitive tool for monitoring *M. tuberculosis *replication [17], as well as active TB disease [18,30].

In the present study we show that both the proportion of IFN-γ and IP-10 positive responders to RD1 selected peptides was significantly lower after successful therapy compared to baseline. It is important to note that the CFP-10 selected peptides induced a stronger and more frequently observed immune response compared to ESAT-6 peptides (data not shown) which emphasizes the need of pooling CFP-10 and ESAT-6 peptides together.

This exploratory study has some limitations. It was conducted on a small number of subjects (17 out of the 41 initially enrolled). The proportion of responders to RD1 selected peptides was higher compared (94%) to previous studies conducted in HIV-uninfected subjects (around 70%) [29,33], and the BCG status of the patients was unknown. BCG coverage in India is high though and we may expect that the majority of the population studied is BCG-vaccinated [46]. Another limitation is related to the cut-off points used to evaluate the response to treatment. These were found by ROC analysis after comparing the results obtained in healthy subjects with patients with active TB before treatment [30,33,36,47]. Consequently they may not be correct when evaluating the response to treatment and greater efforts to find more accurate cut-off points for treatment efficacy should be made. Indeed based on the cut-off used, the assay based on RD1 selected peptides is inferior to the sputum smear as a means to detect failure. However, despite these limitations, the prospective design of the study, the evaluation of 4 *in vitro *assays for TB diagnosis (3 experimental and 1 commercial) and the consistency of the data found between the 2 markers used to evaluate the RD1 responses render the results solid and interesting.

## Conclusions

In conclusion, we are showing (for the first time to our knowledge) that IP-10 response to the QFT-IT antigens might be a useful biomarker for monitoring therapy efficacy in patients with active TB. Similar results were obtained in our previous reports using IFN-γ [18,30]. Therefore, there is no real difference between the two biomarkers (IFN-γ and IP-10) other than the magnitude of the response (greater than 20 fold). Additional studies performed on a larger number of individuals in both high and low burden TB settings are needed to evaluate the consistency of these results.

## List of Abbreviations

AFB: Acid fast Bacilli; BCG: Bacille Calmette-Guerin; CFP-10: Culture filtrate protein-10; ELISA: Enzyme linked immunosorbant assay; ESAT-6: Early secreted antigenic target-6; HIV: Human immunodeficiency virus; HLA: Human leukocyte antigen; IQR: Interquartile range; IFN-γ: Interferon gamma; IP-10: Interferon gamma inducible protein-10; LTBI: Latent tuberculosis infection; PPD: Purified protein derivative; QFT-IT: QuantiFERON-TB Gold In tube; RD: Region of Difference; RNTCP: Revised national TB control programme; T0: Time of diagnosis; T6: End of treatment; TB: Tuberculosis.

## Conflict of interests

DG and EG have European patent N. 1723426 on T-cell assay based on selected RD1 peptides.

## Authors' contributions

Conception and design of the experiments: DG and AR. Study subject recruitment: BR. The experiments were performed by: BSAK. Data acquisition: ST, ML. Data analysis: DG, EG, PHL and AR. Contribution of reagents/materials/analysis tools: DG, AR, ST, ML, GI. Writers of the paper: BSAK and DG. Critical revision of the manuscript: ST, ML, BR, GI. Final approval of the version to be published: BSAK, AR, BR, ST, ML, GI, EG, PHL, DG.

## Pre-publication history

The pre-publication history for this paper can be accessed here:

http://www.biomedcentral.com/1471-2334/11/135/prepub
